# Supplementary material for: Psychotherapeutic Interventions for Depressive Symptoms in Community-Dwelling Older Adults: A Systematic Review with Meta-Analysis
Source: Healthcare (Basel). 2024 Dec 18;12(24):2551. doi: 10.3390/healthcare12242551 (PMC11675262; doi:10.3390/healthcare12242551)
Supplement: Supplementary file 1 [file healthcare-12-02551-s001.zip › Search.pdf]

#	Query	Limiters/Expanders	Last Run Via	Results
S25	S5 AND S14 AND S21 AND S23	Expanders - Apply equivalent subjects<br>Narrow by SubjectAge: - all adult: 19+ years<br>Search modes - Boolean/Phrase	Interface - EBSCOhost Research Databases<br>Search Screen - Advanced Search<br>Database - MEDLINE with Full Text;CINAHL Plus with Full Text;Psychology and Behavioral Sciences Collection	163
S24	S5 AND S14 AND S21 AND S23	Expanders - Apply equivalent subjects<br>Search modes - Boolean/Phrase	Interface - EBSCOhost Research Databases<br>Search Screen - Advanced Search<br>Database - MEDLINE with Full Text;CINAHL Plus with Full Text;Psychology and Behavioral Sciences Collection	409
S23	AB randomised	Expanders - Apply equivalent subjects<br>Search modes - Boolean/Phrase	Interface - EBSCOhost Research Databases<br>Search Screen - Advanced Search<br>Database - MEDLINE with Full Text;CINAHL Plus with Full Text;Psychology and Behavioral Sciences Collection	173,848
S22	S5 AND S14 AND S21	Expanders - Apply equivalent subjects<br>Search modes - Boolean/Phrase	Interface - EBSCOhost Research Databases<br>Search Screen - Advanced Search<br>Database - MEDLINE with Full Text;CINAHL Plus with Full Text;Psychology and Behavioral Sciences Collection	5,711
S21	S15 OR S16 OR S17 OR S18 OR S19 OR S20	Expanders - Apply equivalent subjects<br>Search modes - Boolean/Phrase	Interface - EBSCOhost Research Databases<br>Search Screen - Advanced Search<br>Database - MEDLINE with Full Text;CINAHL Plus with Full	1,724,520

			Text;Psychology and Behavioral Sciences Collection	
S20	home nursing	Expanders - Apply equivalent subjects Search modes - Boolean/Phrase	Interface - EBSCOhost Research Databases Search Screen - Advanced Search Database - MEDLINE with Full Text;CINAHL Plus with Full Text;Psychology and Behavioral Sciences Collection	133,256
S19	home healthcare	Expanders - Apply equivalent subjects Search modes - Boolean/Phrase	Interface - EBSCOhost Research Databases Search Screen - Advanced Search Database - MEDLINE with Full Text;CINAHL Plus with Full Text;Psychology and Behavioral Sciences Collection	66,372
S18	home health care	Expanders - Apply equivalent subjects Search modes - Boolean/Phrase	Interface - EBSCOhost Research Databases Search Screen - Advanced Search Database - MEDLINE with Full Text;CINAHL Plus with Full Text;Psychology and Behavioral Sciences Collection	68,487
S17	home care services	Expanders - Apply equivalent subjects Search modes - Boolean/Phrase	Interface - EBSCOhost Research Databases Search Screen - Advanced Search Database - MEDLINE with Full Text;CINAHL Plus with Full Text;Psychology and Behavioral Sciences Collection	67,966
S16	home care	Expanders - Apply equivalent subjects Search modes - Boolean/Phrase	Interface - EBSCOhost Research Databases Search Screen - Advanced Search Database - MEDLINE	157,241

			with Full Text;CINAHL Plus with Full Text;Psychology and Behavioral Sciences Collection	
S15	community	Expanders - Apply equivalent subjects Search modes - Boolean/Phrase	Interface - EBSCOhost Research Databases Search Screen - Advanced Search Database - MEDLINE with Full Text;CINAHL Plus with Full Text;Psychology and Behavioral Sciences Collection	1,516,513
S14	S6 OR S7 OR S8 OR S9 OR S10 OR S11 OR S12 OR S13	Expanders - Apply equivalent subjects Search modes - Boolean/Phrase	Interface - EBSCOhost Research Databases Search Screen - Advanced Search Database - MEDLINE with Full Text;CINAHL Plus with Full Text;Psychology and Behavioral Sciences Collection	336,616
S13	non-pharmaceutical treatment	Expanders - Apply equivalent subjects Search modes - Boolean/Phrase	Interface - EBSCOhost Research Databases Search Screen - Advanced Search Database - MEDLINE with Full Text;CINAHL Plus with Full Text;Psychology and Behavioral Sciences Collection	237
S12	non-pharmaceutical interventions	Expanders - Apply equivalent subjects Search modes - Boolean/Phrase	Interface - EBSCOhost Research Databases Search Screen - Advanced Search Database - MEDLINE with Full Text;CINAHL Plus with Full Text;Psychology and Behavioral Sciences Collection	2,263
S11	non-pharmaceutical	Expanders - Apply equivalent subjects	Interface - EBSCOhost Research Databases Search Screen -	3,382

		Search modes - Boolean/Phrase	Advanced Search Database - MEDLINE with Full Text;CINAHL Plus with Full Text;Psychology and Behavioral Sciences Collection	
S10	behavior therapy	Expanders - Apply equivalent subjects Search modes - Boolean/Phrase	Interface - EBSCOhost Research Databases Search Screen - Advanced Search Database - MEDLINE with Full Text;CINAHL Plus with Full Text;Psychology and Behavioral Sciences Collection	73,861
S9	psychotherapy, brief	Expanders - Apply equivalent subjects Search modes - Boolean/Phrase	Interface - EBSCOhost Research Databases Search Screen - Advanced Search Database - MEDLINE with Full Text;CINAHL Plus with Full Text;Psychology and Behavioral Sciences Collection	6,523
S8	psychotherapy groups	Expanders - Apply equivalent subjects Search modes - Boolean/Phrase	Interface - EBSCOhost Research Databases Search Screen - Advanced Search Database - MEDLINE with Full Text;CINAHL Plus with Full Text;Psychology and Behavioral Sciences Collection	27,028
S7	cognitive behavioral therapy	Expanders - Apply equivalent subjects Search modes - Boolean/Phrase	Interface - EBSCOhost Research Databases Search Screen - Advanced Search Database - MEDLINE with Full Text;CINAHL Plus with Full Text;Psychology and Behavioral Sciences Collection	64,658

S6	Psychotherapy	Expanders - Apply equivalent subjects Search modes - Boolean/Phrase	Interface - EBSCOhost Research Databases Search Screen - Advanced Search Database - MEDLINE with Full Text;CINAHL Plus with Full Text;Psychology and Behavioral Sciences Collection	229,177
S5	S1 OR S2 OR S3 OR S4	Expanders - Apply equivalent subjects Search modes - Boolean/Phrase	Interface - EBSCOhost Research Databases Search Screen - Advanced Search Database - MEDLINE with Full Text;CINAHL Plus with Full Text;Psychology and Behavioral Sciences Collection	840,311
S4	major depressive disorder	Expanders - Apply equivalent subjects Search modes - Boolean/Phrase	Interface - EBSCOhost Research Databases Search Screen - Advanced Search Database - MEDLINE with Full Text;CINAHL Plus with Full Text;Psychology and Behavioral Sciences Collection	294,956
S3	depressive symptoms	Expanders - Apply equivalent subjects Search modes - Boolean/Phrase	Interface - EBSCOhost Research Databases Search Screen - Advanced Search Database - MEDLINE with Full Text;CINAHL Plus with Full Text;Psychology and Behavioral Sciences Collection	121,914
S2	depressive disorder	Expanders - Apply equivalent subjects Search modes - Boolean/Phrase	Interface - EBSCOhost Research Databases Search Screen - Advanced Search Database - MEDLINE with Full Text;CINAHL Plus with Full Text;Psychology and	326,919

			Behavioral Sciences Collection	
S1	depression	Expanders - Apply equivalent subjects<br>Search modes - Boolean/Phrase	Interface - EBSCOhost Research Databases<br>Search Screen - Advanced Search<br>Database - MEDLINE with Full Text;CINAHL Plus with Full Text;Psychology and Behavioral Sciences Collection	795,270
