# Supplementary material for: Psychotherapeutic Interventions for Depressive Symptoms in Community-Dwelling Older Adults: A Systematic Review with Meta-Analysis
Source: Healthcare (Basel). 2024 Dec 18;12(24):2551. doi: 10.3390/healthcare12242551 (PMC11675262; doi:10.3390/healthcare12242551)
Supplement: Supplementary file 1 [file healthcare-12-02551-s001.zip › Supplementary Material.pdf]

Supplementary Material

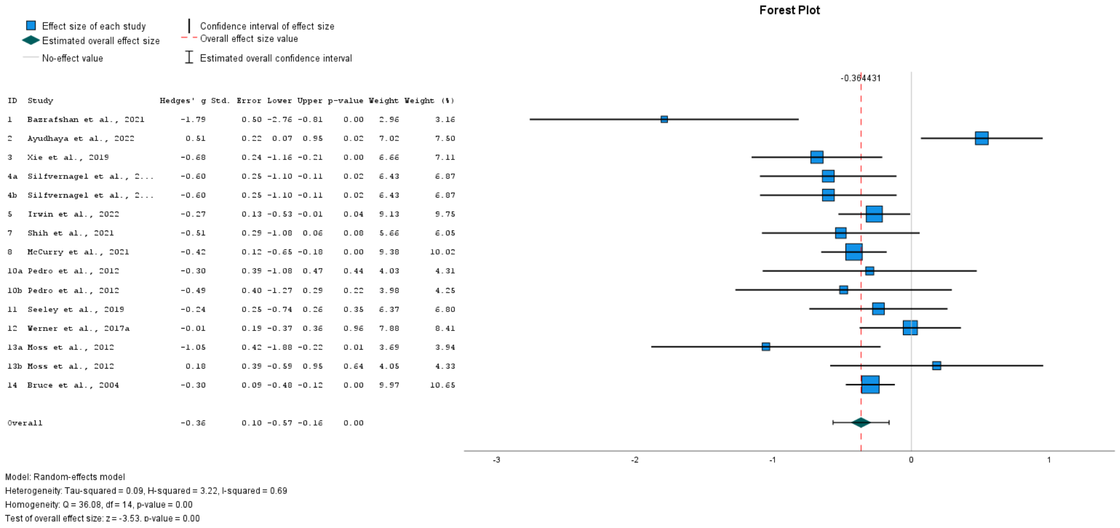

Fig.S1 - Effects of non-pharmacological interventions on depressive symptoms, post-intervention

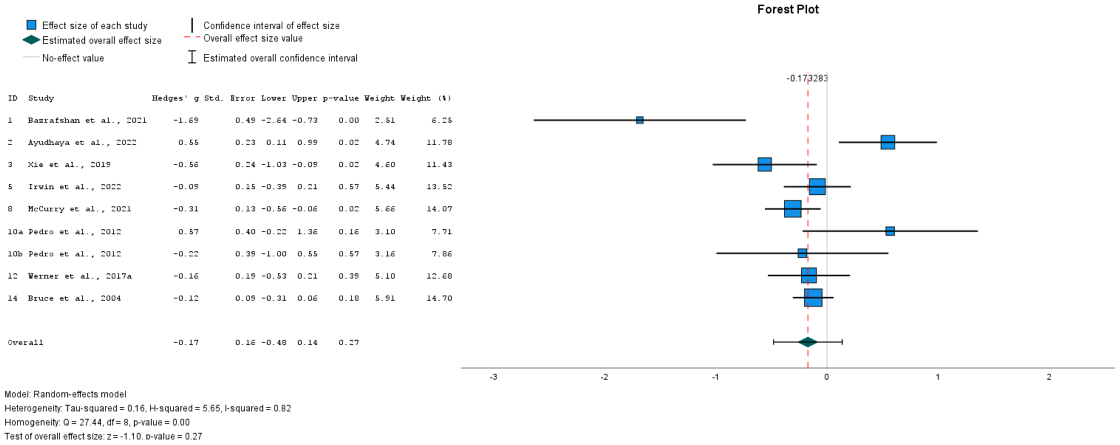

Fig.S2 - Effects of non-pharmacological interventions on depressive symptoms, follow-up

At post-intervention, there was a significant mean combined effect for group non-pharmacological interventions (Hedges‘ adjusted g = -.48, p<.001) and a small non-significant combined effect for individual interventions (Hedges’ adjusted g = -.15, p=.40). There was evidence of homogeneity between subgroups (chi2=2.515, p=.113), Figure 3.

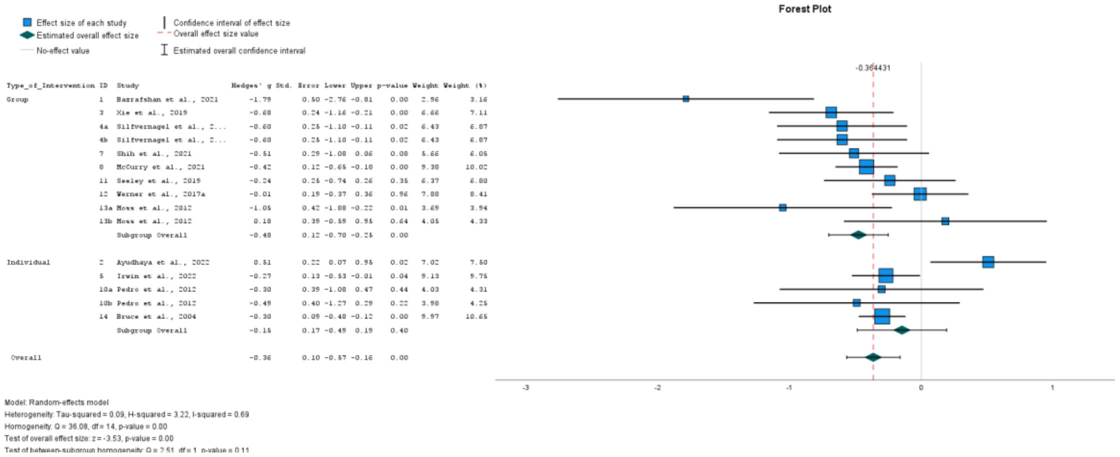

Fig.S3 - Effects of individual and group non-pharmacological interventions

The group intervention with the largest significant effect (large effect) was the ‘Reminiscence Protocol’ (Hedges' adjusted g = -1.79, p<.001, [-2.76, -.81]).

The individual intervention with the largest significant effect (medium effect) was the ‘Prevention of Suicide in Primary Care Elderly’ (Hedges' adjusted g = -.30, p<.001, [-.48, -.12]).

Publication bias

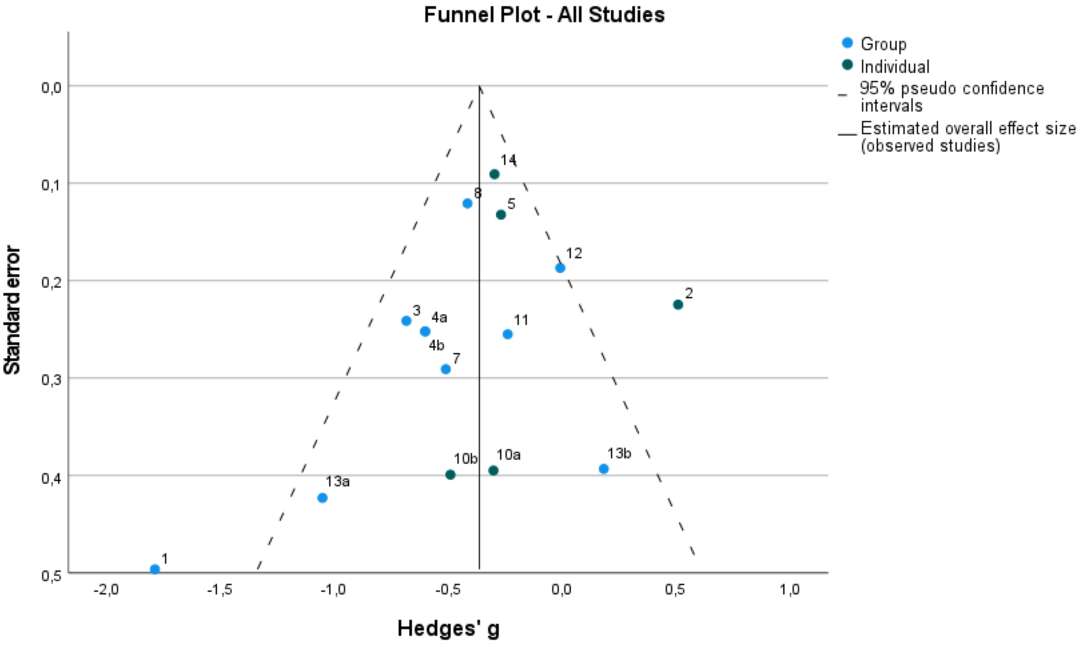

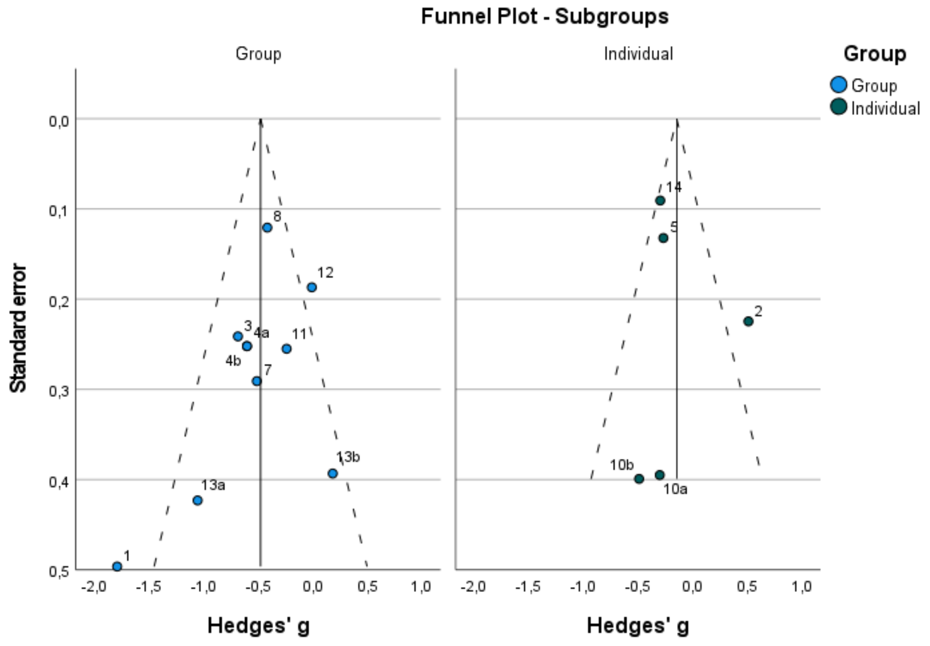

Egger's Regression-Based Test[a]

	Parameter	Coefficient	Std. Error	t	Sig. (2-tailed)	95% Confidence Interval Lower	95% Confidence Interval Upper
Group	(Intercept)	,034	,3133	,109	,916	-,688	,757
	SE[b]	-2,036	1,1726	-1,737	,121	-4,740	,668
Individual	(Intercept)	-,099	,4048	-,245	,822	-1,387	1,189
	SE[b]	-,230	1,6561	-,139	,898	-5,501	5,040
Overall	(Intercept)	-,004	,2475	-,015	,989	-,538	,531
	SE[b]	-1,521	,9481	-1,604	,133	-3,569	,527

a. Random-effects meta-regression

b. Standard error of effect size

The coefficients of the intercepts for all categories (group, individual and general intervention) are not statistically significant ($p>0.05$), so there is no evidence of publication bias.
